# Genome-Wide Copy Number Variations Using SNP Genotyping in a Mixed Breed Swine Population

**DOI:** 10.1371/journal.pone.0133529

**Published:** 2015-07-14

**Authors:** Ralph T. Wiedmann, Dan J. Nonneman, Gary A. Rohrer

**Affiliations:** United States Department of Agriculture, Agricultural Research Service, United States Meat Animal Research Center, Clay Center, Nebraska, United States of America; Huazhong Agricultural University, CHINA

## Abstract

Copy number variations (CNVs) are increasingly understood to affect phenotypic variation. This study uses SNP genotyping of trios of mixed breed swine to add to the catalog of known genotypic variation in an important agricultural animal. PorcineSNP60 BeadChip genotypes were collected from 1802 pigs that combined to form 1621 trios. These trios were from the crosses of 50 boars with 525 sows producing 1621 piglets. The pigs were part of a population that was a mix of ¼ Duroc, ½ Landrace and ¼ Yorkshire breeds. Merging the overlapping CNVs that were observed in two or more individuals to form CNV regions (CNVRs) yielded 502 CNVRs across the autosomes. The CNVRs intersected genes, as defined by RefSeq, 84% of the time – 420 out of 502. The results of this study are compared and contrasted to other swine studies using similar and different methods of detecting CNVR. While progress is being made in this field, more work needs to be done to improve consistency and confidence in CNVR results.

## Introduction

Copy number variation (CNV) refers to segments of DNA typically larger than 1 kb that exist as variable numbers of copies among members of a species. CNV are a form of genetic variation distinct from the more commonly studied single nucleotide polymorphisms (SNP) and CNV have been shown to affect a larger number of nucleotides than SNPs [1]. Many studies have identified CNV in humans [2–4], other model organisms [5,6] and agricultural animals (reviewed in Clop [7]), including pigs [8–21] – the focus of this study. CNVs can affect gene dosage and disrupt normal gene regulation, leading to complex disease traits in humans (reviewed by Stankiewicz and Lupski [22]). In studies in humans, some of the missing heritability of SNP-based GWAS studies of complex traits has been assigned to CNVs [23,24]. The most commonly discussed example of CNV affecting pigs is the white coat phenotype caused by copy number variation of the *KIT* gene [25,26].

CNVs are typically detected using either array comparative genomic hybridization (aCGH) or an SNP genotyping array, although high-throughput sequencing is increasingly being used (reviewed by Kaplan et al. [27]). The main advantage of aCGH is higher signal to noise ratio. However, SNP genotyping chips use less DNA, are less expensive and provide genotyping of the population of animals so that SNP and CNV contributions to the heritability can be simultaneously determined. High-throughput sequencing, given sufficient investment, has superior resolution across the genome, but requires greater computational resources.

Recently published results for detection of CNVs in pigs cover all three methods of detection: aCGH [8, 9, 20], SNP array both with [11,12] and without [13–15, 21] pedigree information, and high-throughput sequencing [16–18]. One study used the SNP array method on 217 highly inbred Iberian pigs and then used high-throughput sequencing on four of those pigs for validation [19]. Most of the pigs studied were either pure or half Chinese breeds, in contrast to the present study which utilizes composite pigs from Landrace, Duroc and Yorkshire lines. Thus, current results may be more relevant to the commercial swine industry. This study uses the Illumina PorcineSNP60 BeadChip (Illumina, San Diego, CA) coupled with the PennCNV algorithm [28]. PennCNV was chosen for this study in part due to its success when compared to competing algorithms [29] and due to its ability to effectively integrate pedigree relationships of boar-sow-offspring trios.

## Results

Every pig had at least one CNV called, the average was 19.9 and the median was 14 CNV called per animal. CNV regions (CNVRs) were determined for the population by merging CNV that overlapped between animals. Including singletons, the full set of 949 CNVR covered 28.8% of the genome. Filtering out the singleton CNV reduced the results to 502 CNVR that cover 19.1% of the genome. The latter number is more consistent with other studies and requiring more than one observation also should eliminate any non-germline CNV as well as many false positives. S1 Table lists the 502 chromosomal positions for each of the CNVR along with their lengths and the number of pigs that contributed to each CNVR. The median number of pigs per CNVR was 8 with a range from 2 to 1129. The lengths of the CNVR ranged from 933 to 31,727,386 bp with a median value of 147,171 bp. The total length of all 502 CNVR is 495.29 Mb.

Table 1 shows the coverage of each chromosome by CNVR, from the low of 3% in chromosome 7 to the high of 61% in chromosome 11. It also lists the total number of CNVR, their average length and the number that intersects known genes as reported by RefSeq [30]. Chromosome 8 exhibited the lowest percentage of CNVR that overlapped genes at 70%, while chromosome 12 had the highest rate of gene overlap at 100%. On an absolute basis, Chromosome 13 had the most CNVR with 63 and the most CNVR that overlapped known genes with 52, slightly ahead of chromosome 1 with 59 and 44, respectively. The total number of RefSeq genes that intersect the CNVRs in this study is 5422, with 1418 being characterized well enough to be assigned gene symbols.

**Table 1:** Summary of the CNVR content of each autosome and the frequency of overlap with genes.

Chr	Length	CNVR length	Coverage	# CNVR	avg length (Kb)	# Genes	% Genes
1	315321320	36925232	0.117	59	629	44	75
2	162569373	37201656	0.229	31	1200	29	94
3	144787320	18957457	0.131	20	948	16	80
4	143465941	5322451	0.037	16	333	12	81
5	111506439	8337930	0.075	21	397	16	76
6	157765591	34634623	0.22	21	16496	16	76
7	134764509	4102732	0.03	18	228	15	83
8	148491824	19480680	0.131	20	974	14	70
9	153670195	44313723	0.288	15	2954	13	87
10	79102372	14048002	0.178	24	585	23	92
11	87690580	53738586	0.613	27	1990	23	85
12	63588570	27230880	0.428	20	1361	20	100
13	218635233	53013240	0.242	63	841	52	83
14	153851968	56829302	0.369	43	1321	36	84
15	157681620	38702927	0.245	42	921	38	90
16	86898990	30933018	0.356	33	937	29	88
17	69701580	7402936	0.106	22	336	19	86
18	61220070	4111482	0.067	7	587	5	71

## Discussion

CNVR have been detected in many species and clearly are important components contributing to the missing heritability of complex traits. This study employed the use of a SNP genotyping beadchip containing 49,208 usable elements spread throughout the genome. Unfortunately, the broad and uneven spacing severely limits the accuracy of predicting end positions of the CNVR, while minimizing false-positives by filtering results to regions spanning three consecutive SNP prevents the identification of many small sized CNVR. Selection of predominantly single locus SNP to include on BeadChips limits the use of this technology to discover CNVR that have copy numbers greater than two. In addition to these technological limits, prior studies in cattle and swine have shown great variation between breeds in CNVR content and a sizable increase in CNVR detection rate for crossbred animals [11, 31].

**Fig 1:**
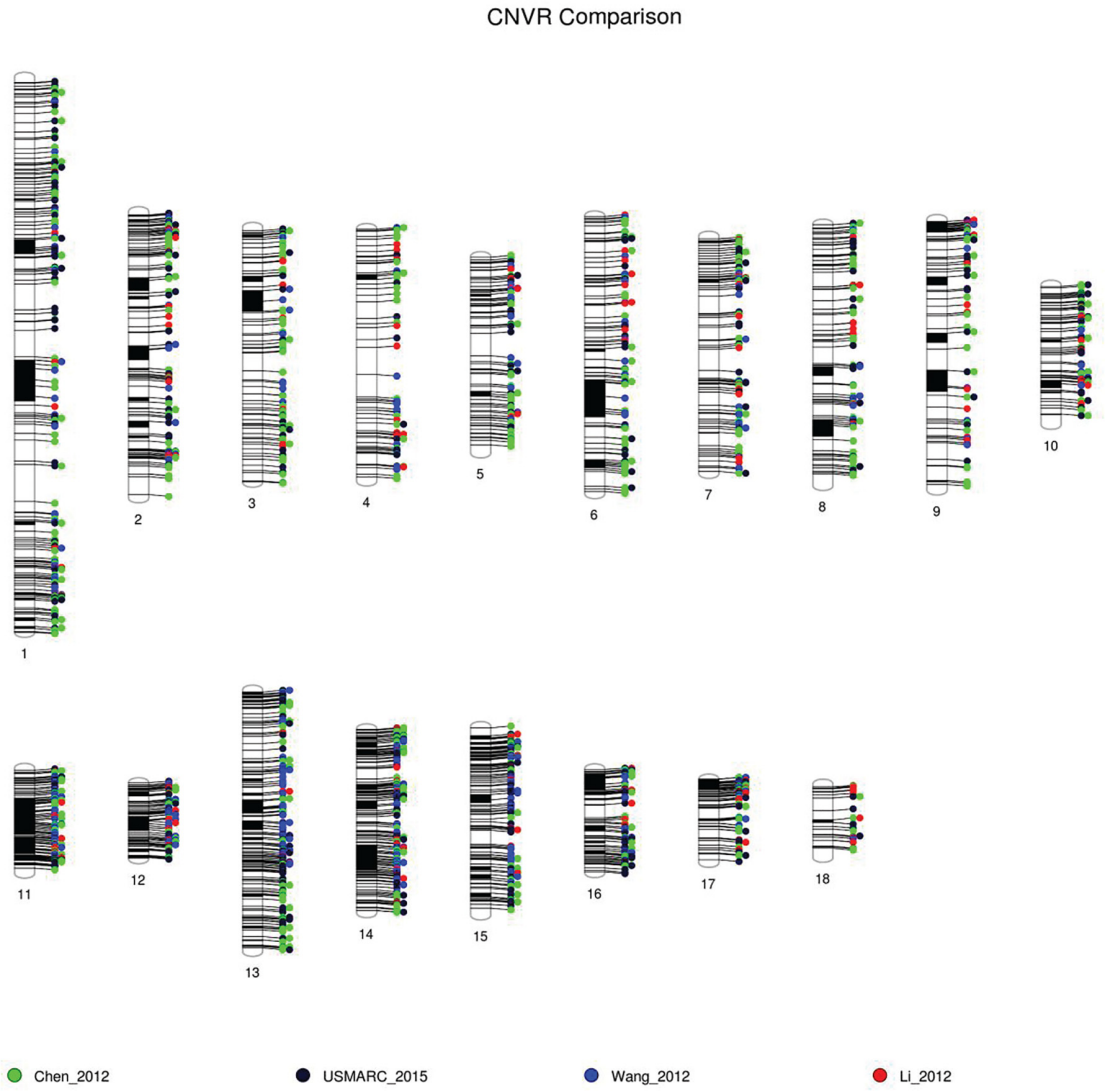
Comparison of CNVR discovered in pigs. Comparison of CNVR discovered with the Illumina SNP60 BeadChip in the current study (USMARC_2015, black) with the results of Chen et al. [12] (Chen_2012, green) and Wang et al. [15] (Wang_2012, blue). In addition, the results of Li et al. [9], which used CGH arrays (Li_2012, red), are also displayed. Diagram was generated using PhenoGram (http://visualization.ritchielab.psu.edu/phenograms/document).

This study uses a mixed breed population with SNP array detection and pedigree information to produce its results. The most similar published studies are those of Wang et al. [15], whose population consisted of 585 pigs that were a cross of Large White and Minzhu and Chen et al. [12] who tested 752 pigs that were an F2 cross of White Duroc and Erhualian. In the same study, Chen et al also reported results for 941 additional pigs covering 17 other populations. In an attempt to find the most robust CNVR that could be used for future investigations, the intersection of CNVR among this study and those of Wang et al. [15] and Chen et al. [12] was determined (Fig 1). Of the 502 CNVR reported in the present study, 237 (47%) overlapped at least one CNVR in the previous studies. There were 48 CNVR (9.6%), some very large, common to both Wang et al. [15] and Chen et al. [12] that overlapped a total of 77 CNVR reported in the present study. The intersection of all three sets of CNVR resulted in 77 regions spanning 12.51 Mb as listed in Table 2. Included in Table 2 is a list of 52 RefSeq genes with a defined gene symbol that intersect the CNVRs.

**Table 2:** CNVR in common across three independent studies.

Chr	Start	End	Overlap	Genes
1	52040272	52093058	1	
1	97924182	97965258	2	PHIP
1	98775053	98820531	3	
1	100050453	100165684	4	
1	172242183	172748312	5	RPSA
1	293928611	293991451	6	
1	294821198	295288944	7	OR7A17
2	13853694	13902607	8	
7	82332463	82456371	9	
8	424993	849384	10	WHSC1, WHSC2
8	110985246	111104427	11	
8	114376195	114671101	12	RPE65
9	3598721	3721967	13	GVIN1, TP53, NLGN1
9	5186145	5500684	14	
9	5757660	5851208	15	
9	85547743	85644823	16	MIOS
11	20495264	20567616	17	
11	26534774	26591544	18	KBTBD6, MTRF1
11	27293298	27433171	19	
11	27718673	27954931	20	
11	27888543	27954931	21	
11	29125223	29212417	22	
11	29592086	29994790	23	OLR1
11	32581919	32878292	24	GABPAP
11	35866787	36037488	25	
11	39815374	40013884	26	FSHB, PPP4R2
11	40927966	40982195	27	
11	43582814	44113334	28	
11	45003730	45537697	29	CSRNP3, PRSS16
11	45857201	45927843	30	
11	46459624	46616903	31	KLHL1
11	49681894	49765012	32	
11	51114485	51188421	33	
12	5078288	5154794	34	RNF157
12	7402572	7651064	35	
12	9877780	10017988	36	NFAT5
12	17351240	17403368	37	CRHR1, RPL13A
12	18614641	18686677	38	KIF18B
12	30823563	31335320	39	S100A16, NLGN1
13	59941517	60165976	40	GXYLT2
13	60647697	60744903	41	PDZRN3
13	68045456	68128761	42	
13	92117925	92262954	43	SLC9A9, CYP39A1
13	92650317	92939983	44	
13	103033108	103194490	45	MME
14	570328	647506	46	
14	2742319	2865914	47	SYK
14	6661819	6747693	48	XPO7, NPM2
14	7363912	7418622	49	BIN3
14	11989764	12229929	50	TRIM35
14	14829391	14922687	51	
14	17250219	17374883	52	
14	18052581	18116518	53	
14	19336577	19467538	54	
14	20698075	20820863	55	
14	21157940	21214238	56	
14	47529479	47801982	57	
14	48731419	48962616	58	
14	50282254	50440156	59	TBC1D10A, SF3A1
14	89402153	89494786	60	
14	99757737	99814376	61	
14	101770612	102244897	62	
14	102629597	102728645	63	
14	103086988	103663341	64	NLRP8
14	106744337	107085009	65	CSTF2T, PRKG1
14	113074497	113136615	66	BTAF1
14	137895546	137953225	67	
14	144641312	144665611	68	
14	144818165	144909224	69	GPR26, GALNT11, CPXM2
15	14002523	14019896	70	
15	15115750	15336530	71	NFAT5, SPOPL, PRSS16
15	17772757	17819850	72	
16	7694530	7700720	73	
16	47536798	47650300	74	NLN, CSPP1
16	73039197	73071316	75	
17	2300488	2345614	76	SGCZ
17	3459317	3510331	77	S100A16

Different statistical methods to discover CNVR from SNP BeadChip data are available and each method produces a unique set of CNVR. Winchester et al. [29] conducted an objective evaluation of different methods using human HapMap data and concluded that the statistical method used should be one developed for the type of data to be analyzed. In addition, they indicated that inclusion of pedigree information in the analyses reduces the number of false-positives. Similarly, Wang et al. [15] analyzed their data with four different software programs and they found that PennCNV yielded the most CNVR that were discovered with at least one of the other programs. As PennCNV is the only software program that incorporates pedigree information with Illumina SNP data, it has been used in all studies with pigs when genotypic data was collected on both parents as well as progeny (trios).

High-throughput sequencing, due to its kilobase resolution, is able to discover the more abundant smaller CNVR. Over 80% of the CNVR discovered by Jiang and coworkers were smaller than the average interval between adjacent SNP on the BeadChip (50 kb) and more than half of the CNVR discovered were between 10 and 20 kb[18]. In the study of Fernández et al. in which sequencing was used on four of the pigs with SNP genotyping data available, they were able to confirm only 16 of 65 BeadChip CNVRs with overlapping high-throughput analysis [19]. To illustrate the differences between BeadChip CNVR and sequencing CNVR, from Table 2 of Fernández et al. [19], CNVR 32 on chromosome 10 is 268 Kb long by BeadChip analysis and is overlapped by 51 smaller CNV found through sequencing. The large spacing of SNP in the Illumina PorcineSNP60 BeadChip and filtering single SNP CNVR creates low resolution CNVR that may be an aggregate of multiple smaller CNVR. The low confirmation rate of BeadChip CNVRs is not due to low resolution, but may be a technical issue related to the design and chemistry of this system. Therefore, stringent criteria need to be applied to limit the number of false-positives reported. Inclusion of pedigree information of genotyped trios and the use of PennCNV reduces the number of false positives. Each study likely finds only a fraction of the CNVR in its population. Poor overlap between swine studies may be due to a high rate of undetected CNVR within each population as well as the dramatically different breeds used in each of the studies.

The high-throughput study of Rubin et al. reported 1928 CNVR in a population of 117 European pigs and wild boars [16]. These CNVR were found to overlap, or nearly overlap, 557 known genes. Of those, only five are in common with the genes listed in Table 2, further indicating an unfortunate lack of consensus between studies. Only 72 genes from Rubin et al. [16] were in common with the 1418 known genes that intersect CNVR observed in the present study Although several studies have successfully reported CNVR in a wide range of swine breeds, insufficient progress has been made in determining the phenotypic effects, and in particular, economically significant effects of these genetic variations. Rubin et al. found few CNVR within regions where signatures of selection were documented [16]. However, their study was based on a comparison between improved and unselected breeds. Two experiments were able to detect significant associations between CNVR and estimated breeding values for boars. Fowler et al. [32] conducted a GWAS for back fat thickness genotyping boars with extremely different breeding values. Along with the GWAS, they also used two different analyses to identify CNVR. Fowler et al. [32] reported 12 different CNVR along with 32 SNP associated with back fat thickness. Revay et al. [33] genotyped boars with extremely high and extremely low breeding values for a fertility trait (direct boar effect on litter size) and reported 35 CNVR detected and seven of these CNVR remained significantly associated with fertility upon testing them in a validation set of animals. However, more detailed studies are required to identify CNVR that affect phenotypic variation within populations.

Failure to identify similar CNVR across studies is concerning. While refinement in experimental protocols is needed, the problem is amplified by variability between breeds and between detection methods. The experiment by Revay et al. [33] utilized purebred boars from the same breeds used to develop the composite population for the current study and 40% of their CNVR associated with fertility were identified in this study. Two of the lines studied for back fat thickness by Fowler et al. [32] were similar to germplasm in this study and 50% of the CNVR associated with back fat thickness were identified in this study. While the primary objective of these two reports was to detect associations with performance, they are the only two studies that used comparable commercially relevant germplasm. More work needs to be done to improve detection techniques for high-throughput testing of animals; thus, facilitating detection of significant CNVR effects on economically important traits.

## Materials and Methods

The experimental procedures were approved and performed in accordance with the U.S. Meat Animal Research Center’s (USMARC) Animal Care and Use committee and the *Guide for Care and Use of Agricultural Animals in Research and Teaching* (FASS, 2010).

### Animals

A composite swine population was developed at the USMARC starting in 2001 by crossing mixed Landrace-Yorkshire sows with one of 24 founding boars – 12 Landrace and 12 Duroc. The second generation was produced by mating Landrace-sired animals to Duroc-sired animals. Subsequent generations were created by choosing one male and ten females produced by each founding boar then randomly mating them while avoiding full-sib and half-sib pairings [34]. This study uses trios from crosses of 50 boars with 525 sows producing 1621 piglets, all born in the years 2005–2010. The piglets were members of the 5^th^ through 8^th^ filial generations of this closed composite population. Animals in this population were managed under typical commercial standards and either sold or slaughtered at the USMARC abattoir using conventional humane stunning methods followed by exsanguination.

### DNA Isolation, SNP Array Genotyping, and Quality Control

Genomic DNA was extracted from the frozen tail sections clipped at 1 day of age of each pig using the Wizard SV Genomic DNA Purification kit (Promega, Madison, WI). The DNA samples were genotyped with the Illumina PorcineSNP60 BeadChip (Illumina, San Diego, CA) [35]. Genotype reactions were completed at the USMARC (Clay Center, NE) and the chips were then scanned at the USDA-ARS Bovine Functional Genomics Laboratory (Beltsville, MD). The scan results were interpreted at the USMARC using Illumina’s BeadStudio Genotyping software.

The SNP with call rates <80% or minor allele frequencies < 0.05 were excluded from the data set, as were SNP that did not map or mapped to multiple positions in the *Sus scrofa* genome assembly 10.2. A final set of 49,208 SNP were used for further analysis.

### Identification of Pig CNVs

Pig CNVs in this study were identified using PennCNV software [28]. PennCNV primarily utilizes the Log R Ratio (LRR) and the B Allele Frequency (BAF) output by BeadStudio, and the population frequency of B allele (PFB) calculated from the genotyping results. To improve the accuracy of the calls, PennCNV was provided a gcmodel file generated by calculating the gc content for the nearest 1 Mb of sequence around each SNP. A minimum of three consecutive SNP was required to call a CNV. PennCNV also utilizes pedigree information to significantly improve the accuracy of CNV calls. This study exclusively used pig samples with full trio information. To further improve the reliability of the results, all CNVs that were called only once in the population were discarded. CNV regions (CNVRs) were created by merging overlapping CNVs.

Mention of trade names or commercial products is solely for the purpose of providing information and does not imply recommendation, endorsement or exclusion of other suitable products by the U.S. Department of Agriculture.

## Supporting Information

S1 Table Information on all CNVR regions discovered. Chromosome position, length, and number of pigs contributing to each of the 502 CNVR identified in the present study.
(XLSX)
